# Binding of SARS-CoV-1/2 NSP1 to DNA Polymerase α‑Primase Inhibits DNA Replication through Reduction of Interaction between DNA and DNA Polymerase α‑Primase

**DOI:** 10.1021/acs.jcim.5c00999

**Published:** 2025-07-30

**Authors:** Hung Van Nguyen, Nguyen Le Ngoc Lan, Mai Suan Li

**Affiliations:** † 86906 Institute of Physics, Polish Academy of Sciences, Al. Lotnikow 32/46, 02-668 Warsaw, Poland; ‡ Biomedical Engineering Department, 598447 University of Technology-VNU HCM, 268 Ly Thuong Kiet Street, Ward 14, District 10, 740500 Ho Chi Minh City, Vietnam

## Abstract

A comprehensive understanding of the atomic level mechanism governing the binding nonstructural protein 1 of SARS-CoV-1 (SARS-CoV-1 NSP1) and SARS-CoV-2 (SARS-CoV-2 NSP1) to Pol α-primase is important to advance the development of small molecule inhibitors for the treatment COVID-19. In this study, we use both all-atom steered molecular dynamics (all-atom SMD) and coarse-grained umbrella sampling (coarse-grained US) simulations to assess the binding affinity of SARS-CoV-1 NSP1 and SARS-CoV-2 NSP1 to Pol α-primase. Our all-atom SMD and coarse-grained US simulations consistently indicate that SARS-CoV-2 NSP1 exhibits stronger affinity for Pol α-primase compared to SARS-CoV-1 NSP1, implying that SARS-CoV-2 poses a greater risk than SARS-CoV-1 in impeding DNA replication for DNA synthesis. Through an energetic decomposition analysis of the interaction energies within these complexes, we identify electrostatic interactions as the primary contributors to the observed difference in binding affinity. We found that hydrogen bonds between Asp33 and Arg616 in SARS1 NSP1-Pol α-primase, and Asp33 with Arg616 and Lys655 in SARS2 NSP1-Pol α-primase, are critical for the interaction of both SARS-CoV-1 NSP1 and SARS-CoV-2 NSP1 with Pol α-primase. Asp33 in SARS-CoV-2 NSP1 shows increased solubility and stability compared to SARS-CoV-1 NSP1, enhancing its association with Pol α-primase. This finding lays the groundwork for innovative strategies aimed at inhibiting the interaction between these entities, offering promising avenues for therapeutic intervention against COVID-19. We also estimated the binding free energy of DNA to Pol α-primase, SARS1 NSP1-Pol α-primase, and SARS2 NSP1-Pol α-primase using the MM-PBSA method. The results show the order: Pol α-primase-DNA < SARS1 NSP1-Pol α-primase-DNA < SARS2 NSP1-Pol α-primase-DNA, indicating that both SARS-CoV-1 NSP1 and SARS-CoV-2 NSP1 reduce DNA binding to Pol α-primase, suggesting impaired DNA synthesis.

## Graphical abstract

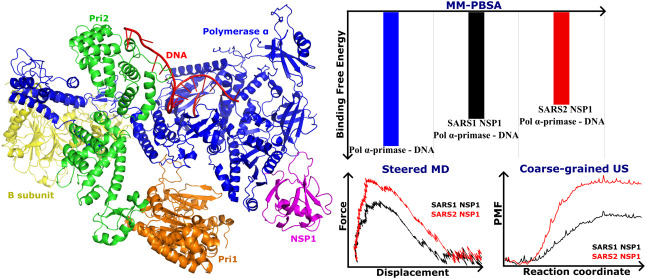

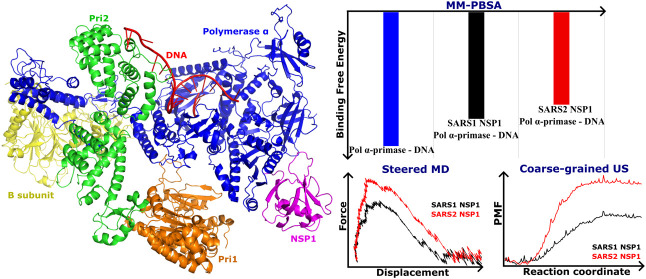

## 1 Introduction

Coronaviruses, characterized by their expansive single-stranded RNA genomes and wide-ranging tropism across mammalian species, have recently gained significant attention. Among them, severe acute respiratory syndrome coronavirus 2 (SARS-CoV-2) emerged as the causative agent of the severe respiratory ailment identified as coronavirus disease 2019 (COVID-19).^1,2^ Demonstrating a high level of transmissibility within human populations, SARS-CoV-2 has precipitated a global pandemic, resulting in around seven million deaths to date. Despite considerable insights into the molecular intricacies of infection and pathogenesis in human cells, the imperative to comprehensively unravel the mechanisms guiding the development of therapeutic agents remains urgent.

SARS-CoV-2 is most closely related to SARS-like coronaviruses isolated from horseshoe bats found in Southeast Asia, sharing ∼79% of its sequence with SARS-CoV-1 and ∼50% with MERS-CoV. Since its initial identification and characterization,^3,4^ genomic sequencing has shown that the SARS-CoV-2 genome encodes from its 5′ to the 3′ end two large polyproteins 1a and 1ab that are cleaved by viral proteases to generate 16 nonstructural proteins (from NSP1 to NSP16), four structural proteins (S, E, M, and N), and a number of other accessory proteins (ORF3a, ORF6, ORF7a/b, ORF8, ORF10, and so on).

Upon viral infection, the host cell promptly initiates an innate immune system response through the interferon (IFN) pathway. This response triggers the secretion of type-I and type-III interferons, activating a signaling cascade that induces the expression of a diverse array of IFN-stimulated genes.^5^ In the context of COVID-19, a distinctive feature is the compromised IFN response in the host, accompanied by elevated levels of inflammatory cytokine production.^6^ SARSCoV-2 employs various mechanisms to dampen the innate immune response, involving the participation of several viral proteins such as NSP1, NSP6, NSP13, M, ORF3a, ORF6, and ORF7a/b.^7,8^

NSP1 comprises 180 amino acids that are arranged into three distinct domains: a N-terminal domain, a linker domain, and an unstructured C-terminal extension.^9^ NSP1 is a constituent of α- and β-coronaviruses but is absent in γ- or δ-coronaviruses. Recognized as a pivotal virulence factor for both SARS and MERS, NSP1 exhibits pleiotropic activities, serving as a potent antagonist against virus- and interferon-dependent signaling and acting as a suppressor of host mRNA translation.^10–13^ Despite exhibiting limited sequence conservation across α- and β-coronaviruses, both effectively induce a robust suppression of host gene expression, potentially through distinct mechanisms.^14–16^ Importantly, α-CoV NSP1 and β-CoV NSP1, despite their divergent sequences, share structural homology in their core domains, adopting a 6-stranded β-barrel structure with an α-helix positioned on the barrel′s rim.^9,17–19^ While the precise significance and functions of NSP1’s globular domain remain incompletely elucidated, recent findings indicate its interaction with the 5′-UTR of SARS-CoV-2 mRNA, facilitating evasion of translational inhibition in infected cells.^20^ Notably, a two-helix hairpin motif near the end of the C-terminal tail in SARS-CoV-2 NSP1 has been identified for its capacity to bind to the 40S ribosomal subunit, obstructing the mRNA entry channel. This structural insight establishes a foundation for understanding the role of NSP1 in impeding host translation within infected cells.^14,21–23^

In recent investigations exploring the human proteins affected by SARS-CoV-2, a noteworthy revelation involves the physical interaction between the N-terminal of SARS-CoV-2 NSP1 and the human primosome.^24,25^ The human primosome comprises DNA polymerase α and primase (called Pol α-primase), orchestrating the initiation of DNA synthesis in DNA replication.^26^ While the nuclear function of DNA polymerase α in genomic duplication is well-established, emerging evidence suggests its potential involvement in innate immunity. Observations of disease-associated mis-splicing in POLA1 substantiate this proposition, the gene encoding the catalytic subunit of DNA polymerase α, leading to aberrant IFN I responses and the manifestation of autoinflammatory phenomena. Although DNA replication and the interferon response are distinct processes, they can be interconnected in the context of viral infections. Viruses that involve DNA replication within host cells may trigger the interferon response as part of the host’s defense mechanism against the viral invasion. The host cell can recognize other viral components and initiate the production of interferons to activate an antiviral state.^27–29^ Additionally, a comprehensive investigation uncovered the biochemical and structural intricacies underlying the interaction between the N-terminal of SARS-CoV-2 NSP1 and Pol α-primase. This revelation strongly indicates that the targeting of Pol α-primase represents a novel mechanism integral to the dynamics of SARS-CoV-2 infection^30^ (Figure 1A).

**Figure 1:**
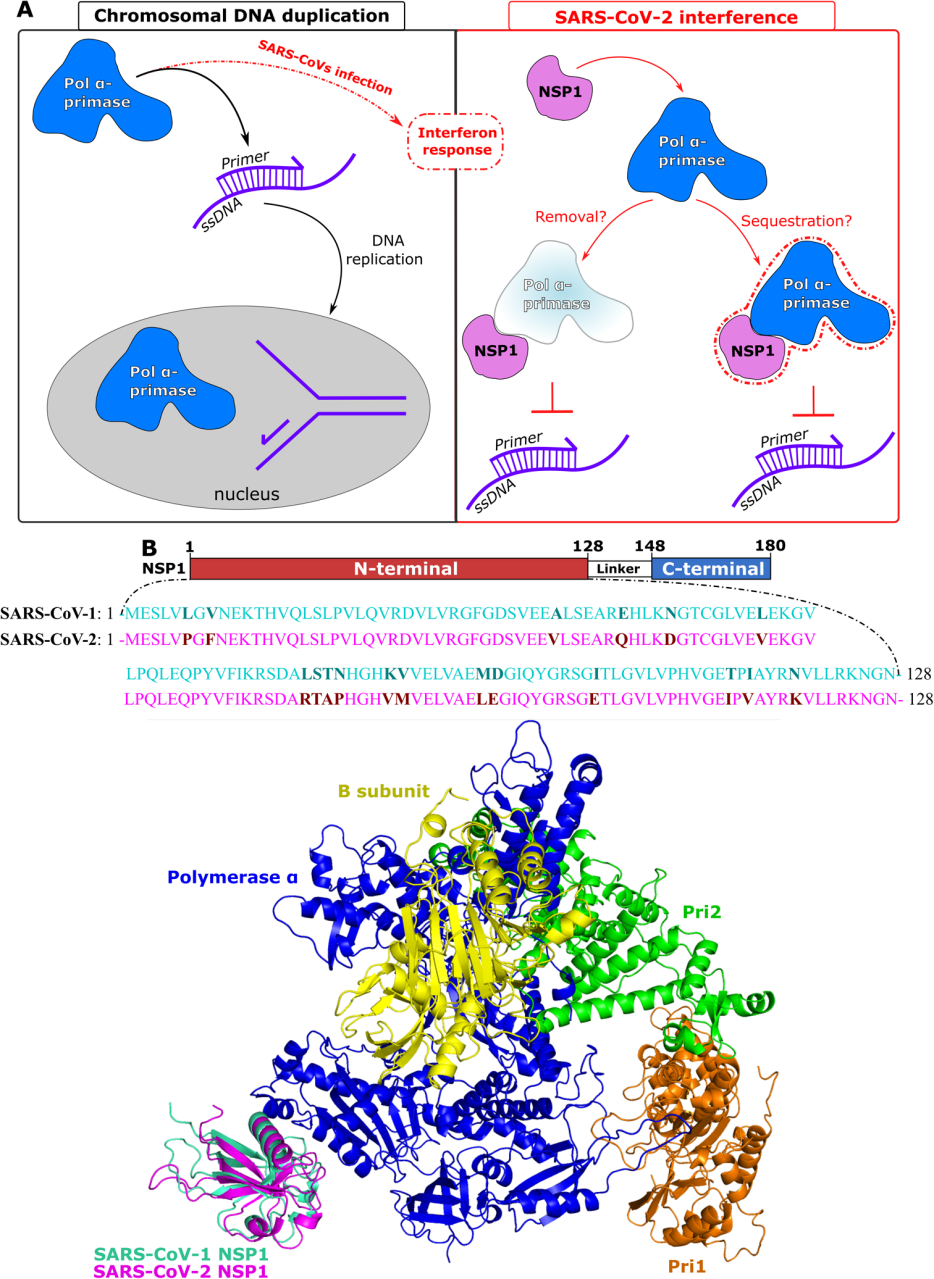
(A) Scheme of (left) beyond its recognized function in chromosomal DNA replication during the S-phase, Pol α-primase has been hypothesized to play a cytoplasmic role in synthesizing RNA/DNA duplexes, potentially influencing the interferon response in the context of viral infections; and (right) the hypothetical mechanisms underlying the NSP1-dependent interference of SARS-CoV-2 with cytoplasmic Pol α-primase. (B) 3D structure of SARS-CoV-1 NSP1 (green-cyan) or SARS-CoV-2 NSP1 (magenta) binds to Pol α-primase, including polymerase α (blue), B subunit (yellow), Pri1 (orange), and Pri2 (green).

Although the experiment has demonstrated the binding of SARS-CoV-2 NSP1 to Pol α-primase and its interference with Pol α’s purported involvement in the immune response during viral infection, the precise mechanism through which this interaction may enhance virulence remains elusive. In this study, we used all-atom steered molecular dynamics (all-atom SMD) and coarse-grained umbrella sampling (coarse-grained US) simulations to access the interaction mechanisms of SARS-CoV-1 NSP1 and SARS-CoV-2 NSP1 to Pol α-promise. Our study’s insights not only shed light on the specific protein epitomes on both SARS-CoV-1 NSP1 and SARS-CoV-2 NSP1 and the catalytic subunits of Pol α-primase but also present potential targets for the development of small molecule inhibitors, thereby paving the way for innovative strategies in inhibiting their interaction. In addition, binding free energy estimations of DNA to Pol α-primase, SARS1 NSP1-Pol α-primase, and SARS2 NSP1-Pol α-primase complexes were calculated using the Molecular mechanics Poisson–Boltzmann surface area (MM-PBSA) method to evaluate the effect of SARS-CoV-1 NSP1 and SARS-CoV-2 NSP1 on the binding between DNA and Pol α-primase.

## 2 Materials and Methods

### 2.1 Preparation of Structures

The 3D structure of SARS-CoV-1 NSP1 bound to Pol α-primase (including DNA polymerase α, B subunit, Pri1, Pri2), namely SARS1 NSP1-Pol α-primase, was taken from Protein Data Bank with ID: 7OPL.^30^ SARS-CoV-2 NSP1 bound to Pol α-primase (called SARS2 NSP1-Pol α-primase) was then constructed by mutating SARS-CoV-1 NSP1 from 7OPL PDB using PYMOL package.^31^ Figure 1B displays the SARS1 NSP1-Pol α-primase and SARS2 NSP1-Pol α-primase structures by using the PYMOL package. Both SARS-CoV-1 NSP1 and SARS-CoV-2 NSP1 interact with only DNA polymerase α.

In addition, to demonstrate that SARS-CoV-1 NSP1 and SARS-CoV-2 NSP1 bind to Pol α-primase and inhibit the DNA synthesis process, we suppose that SARS-CoV-1 NSP1 or SARS-CoV-2 NSP1 binds to Pol α-primase and reduces the binding between DNA and Pol α-primase. To support this hypothesis, we prepared three complexes, including Pol α-primase with DNA (Pol α-primase-DNA), SARS1 NSP1-Pol α-primase with DNA (SARS1 NSP1-Pol α-primase-DNA), and SARS2 NSP1-Pol α-primase with DNA (SARS2 NSP1-Pol α-primase-DNA). The structure of Pol α-primase-DNA was retrieved from Protein Data Bank with ID: 8D9D,^32^ then SARS1 NSP1-Pol α-primase-DNA and SARS2 NSP1-Pol α-primase-DNA were constructed by docking SARS-CoV-1 NSP1 and SARS-CoV-2 NSP1 to their binding site on Pol α-primase-DNA using the HDOCK server.^33^

### 2.2 Conventional Molecular Dynamics Simulations

The complexes were placed in a dodecahedron box with a distance of 1.2 nm between the solute and the box. Their energy was minimized using the steepest descent algorithm followed by a short 2 ns conventional molecular dynamics (CMD) simulation in the NVT and NPT ensembles. Finally, a production CMD simulation was conducted using the leapfrog algorithm^34^ of 1000 ns for the SARS1 NSP1-Pol α-primase and SARS2 NSP1-Pol α-primase complexes and 530 ns for Pol α-primase-DNA, SARS1 NSP1-Pol α-primase-DNA and SARS2 NSP1-Pol α-primase-DNA complexes using CHARMM36 force field^35^ and TIP3P water model.^36^

As can be seen from the RMSD time dependence (Figure S1A), the SARS1 NSP1-Pol α-primase and SARS2 NSP1-Pol α-primase complexes are stable, as the RMSDs reach an equilibrium value after approximately 200 ns, showing fluctuations around 0.55 nm. By applying the clustering analysis to the collected snapshots from a 1000 ns CMD run, we obtained 5 representative structures that is used as initial structures to run 5 independent all-atom SMD simulations. The most populated structure obtained by clustering snapshots collected from 1000 ns CMD simulations for the SARS1 NSP1-Pol α-primase and SARS2 NSP1-Pol α-primase complexes was used to perform coarse-grained US simulations using the MARTINI model.^37^

In addition, Pol α-primase-DNA, SARS1 NSP1-Pol α-primase-DNA and SARS2 NSP1-Pol α-primase-DNA complexes, reached equilibrium after approximately 250 ns as their RMSD saturates displaying fluctuations around 0.5 nm (Figure S1B). Using snapshots recorded in the MD trajectory and the MM-PBSA method,^38^ the binding free energy of these three complexes was calculated.

### 2.3 All-Atom Steered Molecular Dynamics Simulations

All-atom steered molecular dynamics (all-atom SMD) simulation was performed using the CHARMM36 force field^35^ implemented in the GROMACS 2016 package^39^ at 310 K and isotropic pressure of 1 bar, which was obtained using the v-rescale^40^ and Parrinello–Rahman^41^ algorithms, respectively. The TIP3P^36^ water model was used for all systems. Bond lengths were constrained by the parallel linear constraint solver (P-LINCS) algorithm,^42^ allowing a time step of 2 fs. Electrostatic and van der Waals interactions were calculated with a cutoff of 1.2 nm, and the nonbonded interaction pair-list was updated every 10 fs. The Particle Mesh Ewald algorithm^43^ was used to treat long-range electrostatic interactions. Periodic boundary conditions were applied in all directions. The energy of the system was first minimized by using the steepest-descent algorithm, then a short 1 ns MD simulation was then performed for the NVT and NPT ensembles. Finally, we carried out all-atom SMD to pull either SARS-CoV-1 NSP1 or SARS-CoV-2 NSP1 from the binding regions of Pol α-primase. For each system, 5 different trajectories were run at a pulling speeds *v* = 0.5 nm/ns.

A rectangular box with a dimension of 14 × 16 × 35 nm^3^ was used for both complexes. The complexes were immersed in a 0.15 M sodium chloride salt solution and counterions were added to neutralize the system. On one side, a spring with the stiffness k is attached to a dummy atom, which is linked to the C_α_ atom closest to the center of mass (COM) of the binding region of Pol α-primase, encompasses residues forming a contact network with either SARS-CoV-1 NSP1 or SARS-CoV-2 NSP1 (Figure S2). On the other side, the spring is linked to the pulled molecule, which may be either SARS-CoV-1 NSP1 or SARS-CoV-2 NSP1. The pulling direction is parallel to the vector connecting COMs of the binding region of Pol α-primase and either SARS-CoV-1 NSP1 or SARS-CoV-2 NSP1. The complexes were then rotated so that the pulling direction was along the *z*-axis.

The pulling force experienced by a stretched molecule is calculated as follows

1 F=k(Δz−vt)

where *k* is the stiffness of the spring, *v* is the pulling velocity, Δ*z* is the displacement of a real atom connected to the spring in the direction of pulling, respectively. The spring constant *k* was set to 600 kJ/(mol·nm^2^) (≈1020 pN/nm), which is a typical value used in atomic force microscopy (AFM) experiments.^44^

Using the force–displacement profile obtained from all-atom SMD simulations, nonequilibrium work (*W*) then performed by pulling either SARS-CoV-1 NSP1 or SARS-CoV-2 NSP1 from the bound state to the unbound state. It was estimated using the trapezoidal rule

2 $$W=\int Fdz=\sum_{i=1}^{N}\frac{F_{i+1}+F_{i}}{2}\left(z_{i+1}-z_{i}\right)$$

where *N* is the number of simulation steps, *F_i_* and *z_i_* are the force determined by the eq 1 and the position at step *i*, respectively.

To estimate the nonequilibrium binding free energy (Δ*G*), we used Jarzynski’s equality extended to the case when the applied external force grows at a constant speed *v* ^45,46^

3 $$\exp\left(\frac{-\Delta G}{k_{B}T}\right)={\left\langle \exp\left(\frac{W_{t}-\frac{1}{2}k{\left(z_{t}-\mathrm{vt}\right)}^{2}}{k_{B}T}\right)\right\rangle }_{N}$$

here ⟨···⟩*_N_* is the average over *N* trajectories, *z_t_* is the time-dependent displacement, and *W_t_* is the nonequilibrium work at time *t* determined by the eq 2.

From the eq 3, we can extract the equilibrium free energy if the number of simulations is large enough. However, since in our case the pulling is not slow enough and the number of all-atom SMD runs is limited, we can only estimate the nonequilibrium binding and unbinding barriers separating the transition state (TS) from the bound state at *t* = 0 and the unbound state at *t*_end_.^47^

### 2.4 Coarse-Grained Umbrella Sampling Simulations

Since all-atom SMD simulation cannot estimate the equilibrium binding free energy, we used the coarse-grained US simulation to calculate the equilibrium binding free energy of either SARS-CoV-1 NSP1 or SARS-CoV-2 NSP1 to Pol α-primase. In this work, MARTINI 2.2 force field is used to investigate the interaction of SARS-CoV-1 NSP1 and SARS-CoV-2 NSP1 to Pol α-primase, it is accurate enough for extracting the interaction energy for a pair of proteins in an aqueous environment from constraint force profiles. The MARTINI water model was used with a minimum distance between water beads of 1.0 nm.^48^ The system was neutralized by adding sodium chloride salt solution. The temperature was set at *T* = 310 K with a *v*-rescale thermostat,^40^ and pressure was set at *p* = 1.0 bar with a Parinello–Rahman barostat.^41^ Bond lengths in the aromatic amino acid side chains and the bonds between the backbone and side chains were constrained by the P-LINCS algorithm.^42^

To perform coarse-grained US simulations,^49^ for each system, we made a series of configurations along the *z*-axis involving 141 windows each of 0.05 nm. Here the *z*-axis is the reaction coordinate (RC). The choice of the *z*-axis has been already described in all-atom SMD simulation.

To generate the initial configuration for the RC, we pulled either SARS-CoV-1 NSP1 or SARS-CoV-2 NSP1 to the corresponding windows. Then we conducted energy minimization and equilibration using a 3 ns MD simulation restraining the distance between COMs of subsystems. The last snapshot obtained in this simulation will be used as an initial conformation for MD production run.

To hold either SARS-CoV-1 NSP1 or SARS-CoV-2 NSP1 around the center of each window, we applied a bias harmonic potential with a spring constant of 600 kJ/mol/nm^2^. To get a good sampling, for each window, we performed a conventional MD production run of 1000 ns. The WHAM procedure^50^ is then used to determine the one-dimensional potential of mean force (1D-PMF) as a function of the RC.

In addition, we also computed binding free energy (Δ*G*_bind_) of SARS-CoV-1 NSP1 and SARS-CoV-2 NSP1 to Pol α-primase, it is defined as^51,52^

4 $$\Delta G_{\mathrm{bind}}=\left(-k_{B}T\ln\int^{\mathrm{bound}}e^{-G_{1D}\left(r\right)/k_{B}T}\right)-\left({-k}_{B}T\ln\int^{\mathrm{unbound}}e^{-G_{1D}\left(r\right)/k_{B}T}\right)$$

here *G*_1D_(*r*) is the 1D-PMF as a function of *r*, *k*_B_ is the Boltzmann constant, and *T* is the absolute temperature. Symbols ∫^bound^ and ∫^unbound^ refer to summation over bound and unbound regions, respectively. To determine the cutoff distance between the bound and unbound states, we calculated the number interchain contacts as a function of the RC between pulled and nonpulled chains in coarse-grained US simulations. Then the cutoff distance is the distance above which interchain contacts disappear (Figure S3).

### 2.5 MM-PBSA Method

We also applied the MM-PBSA method to estimate the binding free energy of DNA to Pol α-primase, SARS1 NSP1-Pol α-primase, and SARS2 NSP1-Pol α-primase. The binding free energy was calculated using the following equation

5 $$\Delta G_{bind}=\Delta E_{vdW}+\Delta E_{elec}+\Delta G_{polar}+\Delta G_{nonpolar}-T\Delta S$$

Here Δ*E*_elec_ and Δ*E*_vdW_ are the energies of the electrostatic and vdW interactions of DNA with Pol α-primase, SARS1 NSP1-Pol α-primase, and SARS2 NSP1-Pol α-primase. Δ*G*_polar_ is the polar solvation energy, calculated using Delphi software with dielectric constants for solute and solvent of 1 and 80, respectively.^53^ The nonpolar solvate energy Δ*G*_nonpolar_ = γΔSASA, where γ = 0.0072 kcal/mol/nm^2^, and SASA is solvent accessible surface area which was calculated using the gmx sasa tool in the GROMACS with a solvent probe radius of 0.14 nm.^54^ The entropy contribution (*T*Δ*S*) was evaluated using the method proposed by Duan et al.^55^

## 3 Results and Discussion

### 3.1 Binding Affinity of SARS-CoV-2 NSP1 to Pol α-Primase Is Stronger Than SARS-CoV-1 NSP1: All-Atom SMD Simulations

Averaging over 5 independent all-atom SMD runs, the force, pulling work, and free energy barrier profiles of SARS1 NSP1-Pol α-primase and SARS2 NSP1-Pol α-primase are shown in Figure 2 and Table 1.

**Figure 2:**
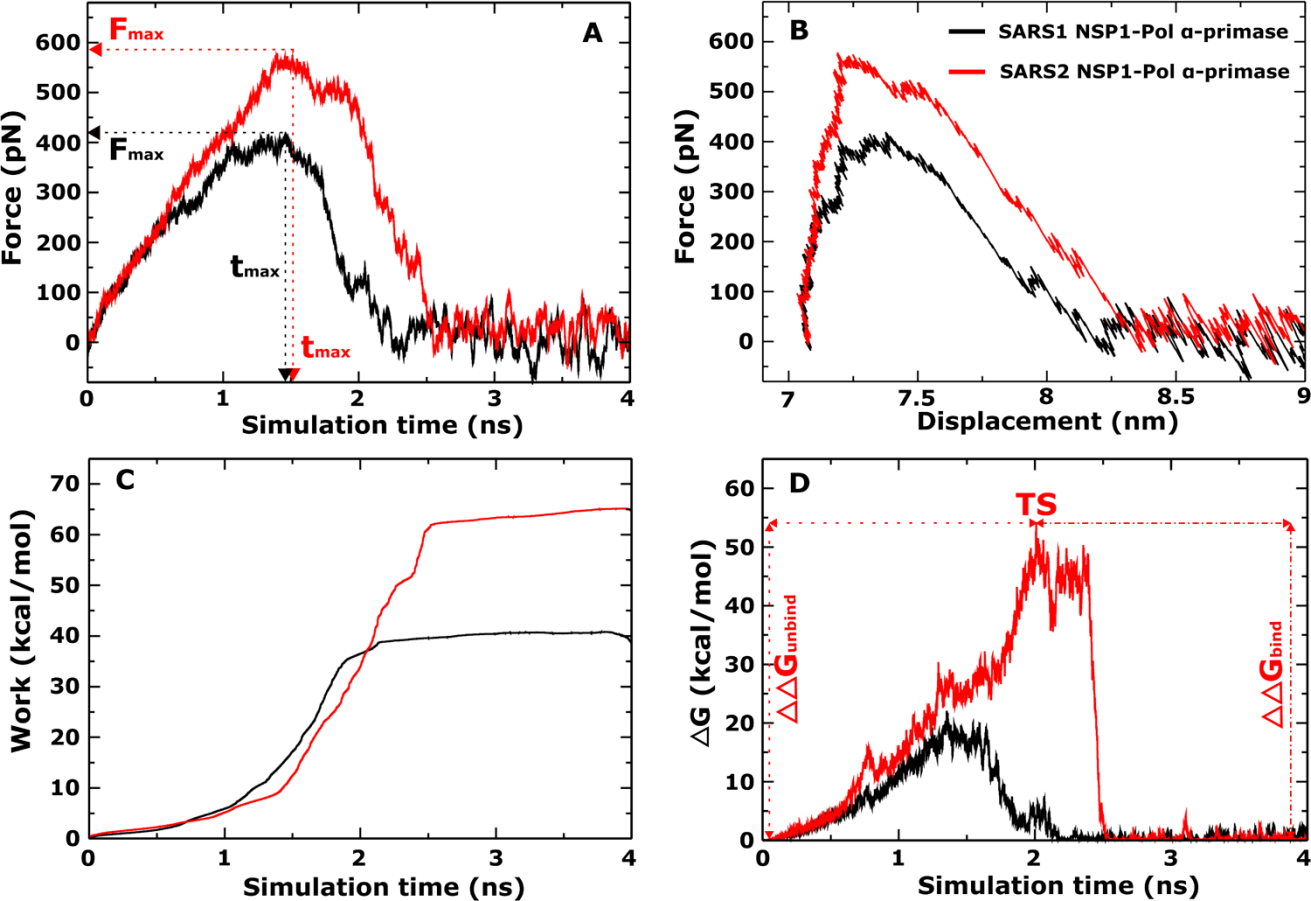
(A) Time dependence of pulling force, (B) displacement dependence of pulling force, (C) time dependence of nonequilibrium work, and (D) time dependence of nonequilibrium energy profiles of the SARS1 NSP1-Pol α-primase and SARS2 NSP1-Pol α-primase complexes. The results were averaged from 5 independent all-atom SMD runs.

**Table 1:** Rupture Force (*F*_max_), Time Rupture (*t*_max_), Non-Equilibrium Work (*W*), Non-Equilibrium Binding Energy Barriers (ΔΔ*G*_bind_ and ΔΔ*G*_unbind_) Were Obtained from 5 Independent SMD Trajectories of SARS1 NSP1-Pol α-Primase and SARS2 NSP1-Pol α-Primase Complexes^a^

	SARS1 NSP1-Pol α-primase	SARS2 NSP1-Pol α-primase
*F*_max_ (pN)	419.95 ± 31.12	578.40 ± 29.41
*t*_max_ (ns)	1.4612 ± 0.1279	1.5629 ± 0.1245
*W* (kcal/mol)	38.36 ± 4.03	64.52 ± 5.44
ΔΔ*G*_unbind_ (kcal/mol)	22.11 ± 4.14	54.18 ± 4.52
ΔΔ*G*_bind_ (kcal/mol)	21.53 ± 3.92	53.14 ± 4.84

a Here the errors represent standard deviations.

The force-time and force–displacement profiles of the two complexes show a marked difference in binding strength between SARS-CoV-1 NSP1 (*F*_max_ = 419.95 ± 31.12 pN) and SARS-CoV-2 NSP1 (*F*_max_ = 578.40 ± 29.41 pN) to Pol α-primase (Figure 2A,2B and Table 1). This indicates that SARS-CoV-2 NSP1 binds more strongly to Pol α-primase than SARS-CoV-1 NSP1.

Since the nonequilibrium work (*W*) appears to be a more reliable indicator for characterizing the relative binding affinity than *F*_max_, a detailed study of *W* is necessary. The work exhibited rapid increase until the pulled molecule (SARS-CoV-1 NSP1 and SARS-CoV-2 NSP1) dissociated from the binding region, stabilizing when the interaction between the two subsystems ceased (Figure 2C). For both SARS1 NSP1-Pol α-primase and SARS2 NSP1-Pol α-primase, we obtained W values of 38.36 ± 4.03 kcal/mol and 64.52 ± 5.44 kcal/mol, respectively (Table 1). Consequently, in alignment with the findings related to *F*_max_, this result further substantiates the assertion that SARS-CoV-2 NSP1 exhibits greater activity than SARS-CoV-1 NSP1, thereby enhancing their binding strength to Pol α-primase.

Figure 2D illustrates the time dependence of the nonequilibrium binding free energy barrier (Δ*G*) estimated using the eq 3 for these complexes. The peak corresponds to the transition state (TS) with Δ*G* = Δ*G*_TS_. At the initiation of the bound state, we have Δ*G*_bound_ = Δ*G*(*t*_0_ = 0) ≈ 0 kcal/mol, whereas the unbound state is observed at the end of the simulation also with Δ*G*_unbound_ = Δ*G*(*t*_end_) ≈ 0 kcal/mol. Consequently, the binding and unbinding free energy barriers, denoted as ΔΔ*G*_bind_ = Δ*G*_TS_ – Δ*G*_unbound_ and ΔΔ*G*_unbind_ = Δ*G*_TS_ – Δ*G*_bound_, respectively, exhibit a roughly equal magnitude. For both SARS1 NSP1-Pol α-primase and SARS2 NSP1-Pol α-primase, we obtained the ΔΔ*G*_unbind_ values of 22.11 ± 4.14 and 54.18 ± 4.52 kcal/mol, and the ΔΔ*G*_bind_ values of 21.53 ± 3.92 and 53.14 ± 4.84 kcal/mol, respectively (Table 1). These results provide additional support for the conclusion that SARS-CoV-2 NSP1 forms a tighter bond with Pol α-primase compared to SARS-CoV-1 NSP1. It should be noted that the equality of nonequilibrium free energy barriers of bound and unbound states is an artifact caused by the too low statistical sample size of only 5 pull-out trajectories, especially for the rather large complexes studied here (approximately 40,000 atoms). However, in this study, we focused on the relative free energy of binding, i.e., comparing the binding affinity of SARS-CoV-1 NSP1 and SARS-CoV-2 NSP1 to the Pol α-primase complex. Our results show that even if the systems have not yet reached equilibrium, we can obtain the correct trend for the relative binding affinity, while saving a lot of computational resources.

In summary, given the stronger binding ability of SARS-CoV-2 NSP1 to Pol α-primase compared to SARS-CoV-1 NSP1, we hypothesize that SARS-CoV-2 poses a greater threat than SARS-CoV-1 by interfering with DNA replication for DNA synthesis.

### 3.2 Binding of SARS-CoV-1 NSP1 and SARS-CoV-2 NSP1 to Pol α-Primase Is Driven by Electrostatic Interactions

The temporal evolution of the van der Waals (vdW), electrostatic, and total interaction (electrostatic + vdW) energies of the SARS1 NSP1-Pol α-primase and SARS2 NSP1-Pol α-primase complexes in the solvent environment is presented in Figure 3 and Table 2. Based on the average results of 5 all-atom SMD runs, it can be concluded that the interaction energy between SARS-CoV-2 NSP1 and Pol α-primase is more negative than that of SARS-CoV-1 NSP1, implying greater stability for SARS2 NSP1-Pol α-primase compared to its SARS-CoV-1 NSP1 counterpart.

**Figure 3:**
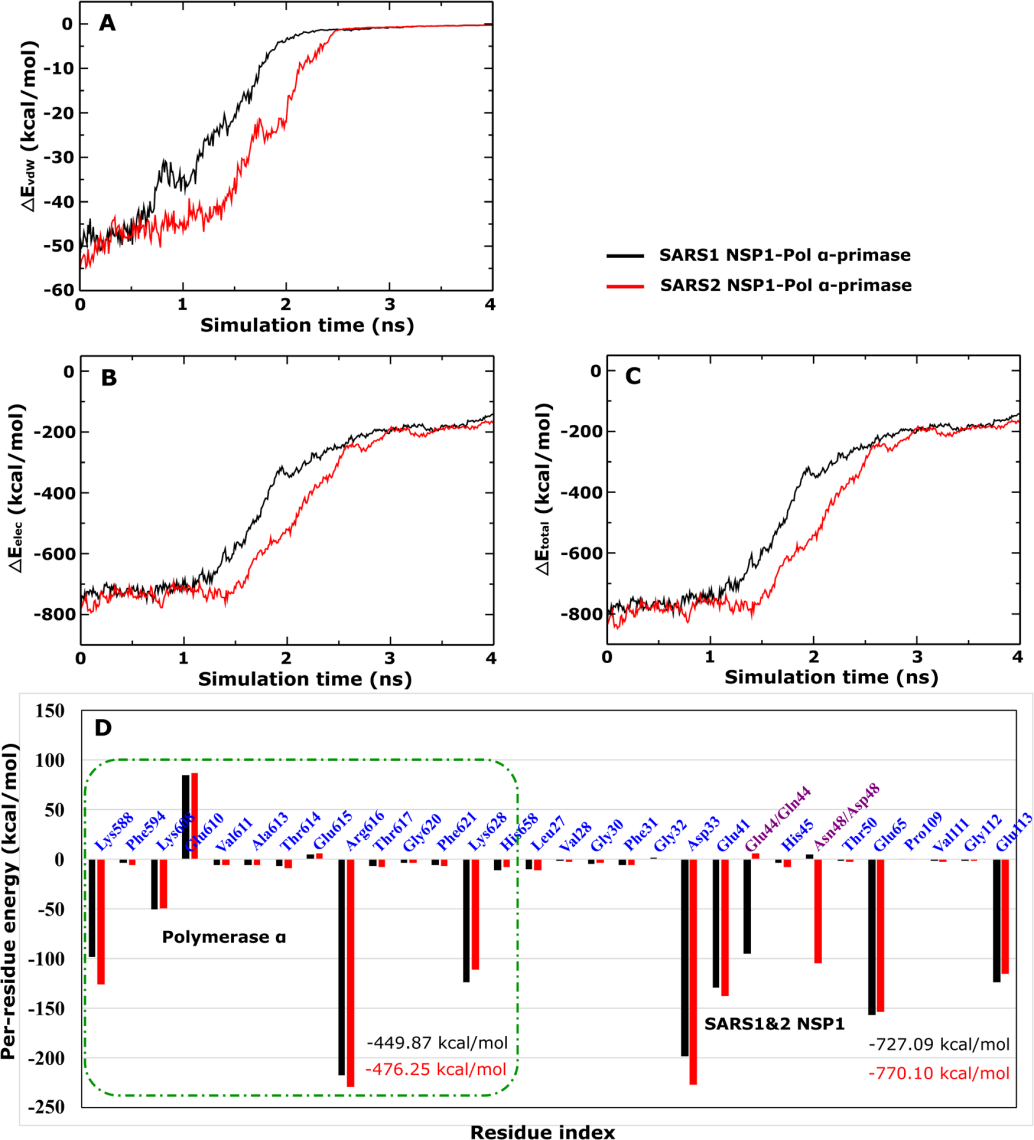
Time dependence of (A) vdW interaction energy, (B) electrostatic interaction energy, (C) total nonbonded interaction energy, and (D) total nonbonded interaction energy of per-residue at the binding regions for a [0–*t*_max_] time window of SARS1 NSP1-Pol α-primase (black) and SARS2 NSP1-Pol α-primase (red). The results were averaged from 5 independent all-atom SMD runs.

**Table 2:** Non-Bonded Interaction Energies (kcal/mol) of SARS1 NSP1-Pol α-Primase and SARS2 NSP1-Pol α-Primase^a^

	SARS1 NSP1-Pol α-primase	SARS2 NSP1-Pol α-primase
ΔE_vdW_	–38.41 ± 2.62	–46.90 ± 1.05
Δ*E*_elec_	–707.89 ± 11.34	–737.51 ± 6.93
Δ*E*_total_	–746.30 ± 13.96	–784.41 ± 7.98

a The results were obtained for a [0–*t*_max_] time window and averaged from 5 all-atom SMD trajectories. Here the errors represent standard deviations.

The vdW energy fluctuates around ∼−52 kcal/mol at *t* = 0 ns and then converges to 0 kcal/mol after 2.5 ns for both SARS1 NSP1-Pol α-primase and SARS2 NSP1-Pol α-primase (Figure 3A). Although SARS-CoV-1 NSP1 and SARS-CoV-2 NSP1 carry the same charge of −2*e* and interact with Pol α-primase (+3*e*) (Table S1), the electrostatic energy of SARS-CoV-2 NSP1 toward Pol α-primase shows a more negative trend compared to SARS-CoV-1 NSP1. This is due to the different contributions of interaction energies associated with each residue in the binding regions of both SARS1 NSP1-Pol α-primase and SARS2 NSP1-Pol α-primase, as described below.

Initially the electrostatic energy of both complexes starts at ∼−750 kcal/mol and then stabilizes around ∼−200 kcal/mol after 2.5 ns (Figure 3B). The difference between the total interaction energy and the electrostatic energy is negligible (see Figure 3C), clearly showing that the contribution of electrostatic energy plays a more important role than the vdW energy in the binding of both SARS1 NSP1-Pol α-primase and SARS2 NSP1-Pol α-primase. Furthermore, averaging the results over 5 trajectories within the [0, *t*_max_] time window in all-atom SMD simulations, we observe a noticeable difference in strength between Δ*E*_elec_ and Δ*E*_vdW_ in both heterodimers (Table 2). For SARS1 NSP1-Pol α-primase, Δ*E*_elec_ is −707.89 ± 11.34 kcal/mol, which is significantly lower than Δ*E*_vdW_ = −38.41 ± 2.62 kcal/mol. Similarly, for SARS2 NSP1-Pol α-primase, Δ*E*_elec_ = −737.51 ± 6.93 kcal/mol is clearly more negative compared to Δ*E*_vdW_ = −46.90 ± 1.05 kcal/mol. Both Δ*E*_elec_ and Δ*E*_vdW_ of SARS2 NSP1-Pol α-primase are smaller in magnitude compared to SARS1 NSP1-Pol α-primase. Therefore, Δ*E*_total_ for SARS2 NSP1-Pol α-primase (−784.41 ± 7.98 kcal/mol) exhibits a more negative value than that of SARS1 NSP1-Pol α-primase (−746.30 ± 13.96 kcal/mol), indicating that SARS-CoV-2 NSP1 displays stronger binding to Pol α-primase compared to SARS-CoV-1 NSP1.

In addition, to ensure that electrostatic energy has a more prominent role than vdW energy under physiological conditions, we also calculated the nonbonded interaction energies in the presence of explicit solvent over a 250 ps average at the bound state from 5 all-atom SMD trajectories. As described in Table S2 (SI), the electrostatic energy (Δ*E*_elec_ = −3325.17 ± 14.12 and −3353.68 ± 13.92 kcal/mol for SARS1 NSP1-Pol α-primase and SARS2 NSP1-Pol α-primase, respectively) remains considerably more negative than the vdW energy (Δ*E*_vdW_ = −237.98 ± 1.94 and −253.24 ± 1.17 kcal/mol for SARS1 NSP1-Pol α-primase and SARS2 NSP1-Pol α-primase, respectively) for both SARS-CoV-1 NSP1 and SARS-CoV-2 NSP1 binding to the Pol α-primase complex, reinforcing the conclusion that electrostatic energy is the main factor driving the binding of both NSP1 variants to the complex.

### 3.3 Role of Specific Residues in the Binding Regions of SARS1 NSP1-Pol α-Primase and SARS2 NSP1-Pol α-Primase Complexes

To indicate the contribution of residues at the binding regions for these complexes, we computed the per-residue interaction energy in the bound state at the binding regions. This calculation considered the images collected within the window [0, *t*_max_] and was derived from the average from 5 all-atom SMD trajectories. The contribution of the residues within the binding regions of both complexes is depicted in Figure 3D. We assume that important residues must have an interaction energy with the absolute value of which exceeds 25 kcal/mol.

For SARS1 NSP1-Pol α-primase, the total interaction energy of all residues at the binding region of SARS-CoV-1 NSP1 amounts to approximately −727.09 kcal/mol, significantly more than Pol α-primase of −449.87 kcal/mol. This is derived from the contribution of charged residues of SARS-CoV-1 NSP1 (Asp33 (−198.20 kcal/mol), Glu41 (−129.68 kcal/mol), Glu44 (−95.54 kcal/mol), Glu65 (−156.81 kcal/mol), and Glu113 (−123.73 kcal/mol)), as well as from charged residues of DNA polymerase α (Lys588 (−98.80 kcal/mol), Lys608 (−50.63 kcal/mol), Glu610 (84.75 kcal/mol), Arg616 (−217.53 kcal/mol), and Lys628 (−124.42 kcal/mol)). Consequently, the binding of SARS1 NSP1-Pol α-primase is primarily driven by the charged residues. Most positively charged residues at the binding region of SARS1 NSP1-Pol α-primase stabilize the complex with negative energy, while only the negatively charged Glu610 residue of Pol α-primase destabilizes it with positive energy.

For SARS2 NSP1-Pol α-primase, residues contributing to total interaction energy at the binding regions appear to be consistent between SARS-CoV-2 NSP1 and Pol α-primase, except for the difference observed between Glu44 (−95.54 kcal/mol) of SARS-CoV-1 NSP1 and Asp48 (−105.12 kcal/mol) of SARS-CoV-2 NSP1. Specifically, the charged residues at the binding region of SARS-CoV-2 NSP1 (Asp33 (−227.09 kcal/mol), Glu41 (−137.89 kcal/mol), Asp48 (−105.12 kcal/mol), Glu65 (−154.21 kcal/mol), and Glu113 (−114.91 kcal/mol)), along with the charged residues at the binding region of Pol α-primase (Lys588 (−126.62 kcal/mol), Lys608 (−49.05 kcal/mol), Glu610 (87.49 kcal/mol), Arg616 (−229.03 kcal/mol), and Lys628 (−111.61 kcal/mol)), are the primary contributors. These results in a more negative total interaction energy of all residues at the binding region of SARS2 NSP1-Pol α-primase (−770.10 kcal/mol for SARS-CoV-2 NSP1, and −476.25 kcal/mol for Pol α-primase) compared to SARS1 NSP1-Pol α-primase.

Taken together, the stability of both complexes is predominantly determined by positively charged residues located at their binding regions. Notably, the charged residues at the binding region of SARS2 NSP1-Pol α-primase contribute to a more negative interaction energy compared to that observed in SARS1 NSP1-Pol α-primase.

### 3.4 Key Hydrogen Bond Pairs Maintain Binding Affinities of SARS-CoV-1 NSP1 and SARS-CoV-2 NSP1 to Pol α-Primase

To delineate the crucial hydrogen bond pairs responsible for upholding the binding affinity between SARS-CoV-1 NSP1 and SARS-CoV-2 NSP1 to Pol α-primase, we conducted an analysis of hydrogen bond networks and the occupancy of such pairs in the SARS1 NSP1-Pol α-primase and SARS2 NSP1-Pol α-primase complexes. This analysis, detailed in Figure 4 and Table S3, involved the calculation of hydrogen bond networks at specific intervals relative to the *F*_max_ during all-atom SMD simulations. Specifically, snapshots capturing the hydrogen bond networks were gathered at distances of 0.1 nm preceding and 0.15 nm following *F*_max_, as determined from the displacement-time profile derived from each of the five trajectories of all-atom SMD simulations (Figure 4). The occupancy of hydrogen bond pairs, essential for maintaining the stability of the complexes, was assessed by averaging their presence within the aforementioned distance range surrounding *F*_max_, as indicated by the displacement-time profiles obtained for each of the five trajectories of SARS1 NSP1-Pol α-primase and SARS2 NSP1-Pol α-primase simulations (Table S3).

**Figure 4:**
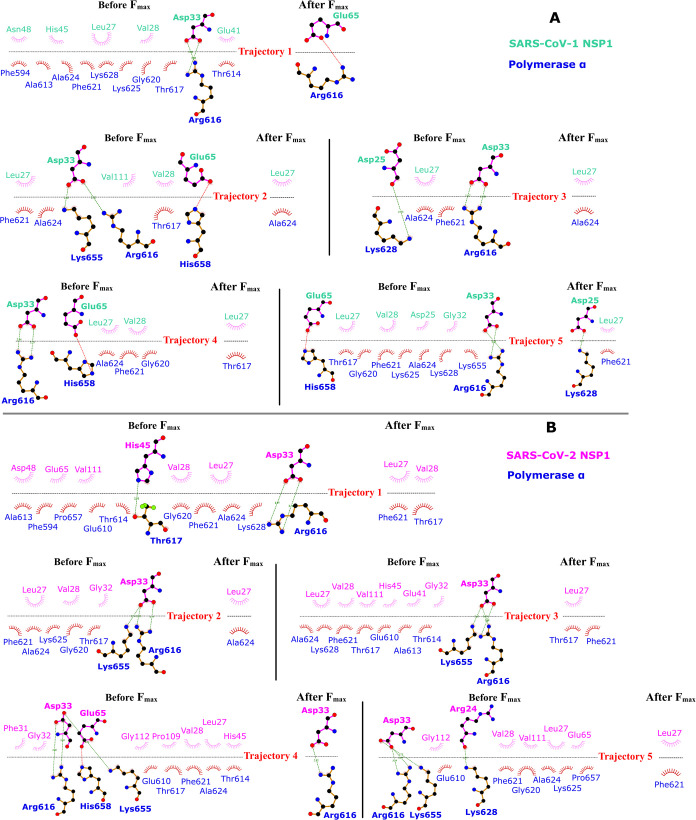
Hydrogen bond networks of (A) SARS1 NSP1-Pol α-primase and (B) SARS2 NSP1-Pol α-primase for 5 trajectories of all-atom SMD simulations for before and after *F*_max_. Snapshots were taken at a distance of 0.1 nm before and 0.15 nm after *F*_max_, based on the displacement-time profile. These results generated by the LigPlot.^56^

Figure 4 shows that there were not only some pairs of hydrogen bonds but also a few residue pairs participating in hydrophobic interactions of SARS-CoV-1 NSP1 and SARS-CoV-2 NSP1 to Pol α-primase. Here, we consider only the hydrogen bonds, which play a major role in maintaining SARS1 NSP1-Pol α-primase and SARS2 NSP1-Pol α-primase configurations. In detail, for SARS1 NSP1-Pol α-primase, there were four hydrogen bond pairs of Asp33::Arg616, Asp33::Lys655, Glu65::His658, and Asp25::Lys628 found in the snapshots before *F*_max_, whereas only Glu65::Arg616 and Asp25::Lys628 were observed after *F*_max_. Importantly, the hydrogen bond pair Asp33::Arg616 was consistently observed in all five trajectories for SARS1 NSP1-Pol α-primase, forming two hydrogen bonds. Additionally, in SARS1 NSP1-Pol α-primase, the pairs Glu65::His658 and Asp25::Lys628 appeared in three and two trajectories, respectively, before reaching *F*_max_. In the case of SARS2 NSP1-Pol α-primase, five hydrogen bond pairs—Asp33::Arg616, Asp33::Lys655, His45::Thr617, Glu65::His658, and Arg24::Lys628—were identified in snapshots taken before *F*_max_, with only Asp33::Arg616 persisting after *F*_max_. Notably, Asp33 was involved in two hydrogen bonds with Arg616 and one with Lys655, consistently observed across all five trajectories before reaching *F*_max_.

Table S3 presents the occupancy data for hydrogen bond pairs in the complexes of SARS1 NSP1-Pol α-primase and SARS2 NSP1-Pol α-primase. In this analysis, hydrogen bond pairs with an occupancy greater than 20% were considered to significantly contribute to the maintenance of the respective complex configurations. Specifically, in SARS1 NSP1-Pol α-primase, four hydrogen bond pairs—Asp33::Arg616, Asp33::Lys655, Glu65::His658, and Asp25::Lys628—exhibited occupancies exceeding 20% while SARS2 NSP1-Pol α-primase displayed significant occupancy for four hydrogen bond pairs—Asp33::Arg616, Asp33::Lys655, His45::Thr617, and Arg24::Lys628. Significantly, Asp33::Arg616 emerged as a pivotal factor in maintaining the binding affinity of SARS1 NSP1-Pol α-primase. Meanwhile, in the context of SARS2 NSP1-Pol α-primase, the hydrogen bond pairs Asp33::Arg616 and Asp33::Lys655 were identified as key contributors to the maintenance of binding affinity.

In summary, while multiple hydrogen bond pairs contribute to the binding between SARS-CoV-1 NSP1 and SARS-CoV-2 NSP1 to Pol α-primase, those formed between Asp33 and Arg616 in SARS1 NSP1-Pol α-primase, and Asp33 to Arg616 and Lys655 in SARS2 NSP1-Pol α-primase, emerged as crucial factors driving the interaction of both SARS-CoV-1 NSP1 and SARS-CoV-2 NSP1 with Pol α-primase. Importantly, the presence of Asp33 in SARS-CoV-2 NSP1 confers higher solubility and stability in binding to Pol α-primase compared to SARS-CoV-1 NSP1. This disparity in binding attributes is accountable for the observed differences between the interactions of SARS-CoV-1 NSP1 and SARS-CoV-2 NSP1 with Pol α-primase.

### 3.5 Binding Affinities of SARS-CoV-1 NSP1 and SARS-CoV-2 NSP1 to Pol α-Primase: Coarse-Grained US Simulations

The MARTINI coarse-grained US simulation was employed for the determination of the absolute binding affinities of both SARS1 NSP1-Pol α-primase and SARS2 NSP1-Pol α-primase. To confirm the attainment of equilibrium, we computed the 1D-PMF for three distinct time intervals: [50–500 ns], [50–800 ns], and [50–1000 ns]. As illustrated in Figure S4, the 1D-PMF profiles across these intervals exhibited substantial similarity, indicating that our data had reached equilibration. Consequently, the 1D-PMF profile derived from the longest time window was utilized for the estimation of binding free energy (Δ*G*_bind_) of SARS1 NSP1-Pol α-primase and SARS2 NSP1-Pol α-primase (Figure 5). To extract the Δ*G*_bind_ from the 1D-PMF profiles, we have to estimate the cutoff distance between bound and unbound states, as described in “Section 2”. From the distance dependence of the number of interchain contacts (Figure S3), we obtained the cutoff distance of 4.90 and 4.20 nm for SARS1 NSP1-Pol α-primase and SARS2 NSP1-Pol α-primase, respectively. The Δ*G*_bind_ is then estimated by using the eq 4.

**Figure 5:**
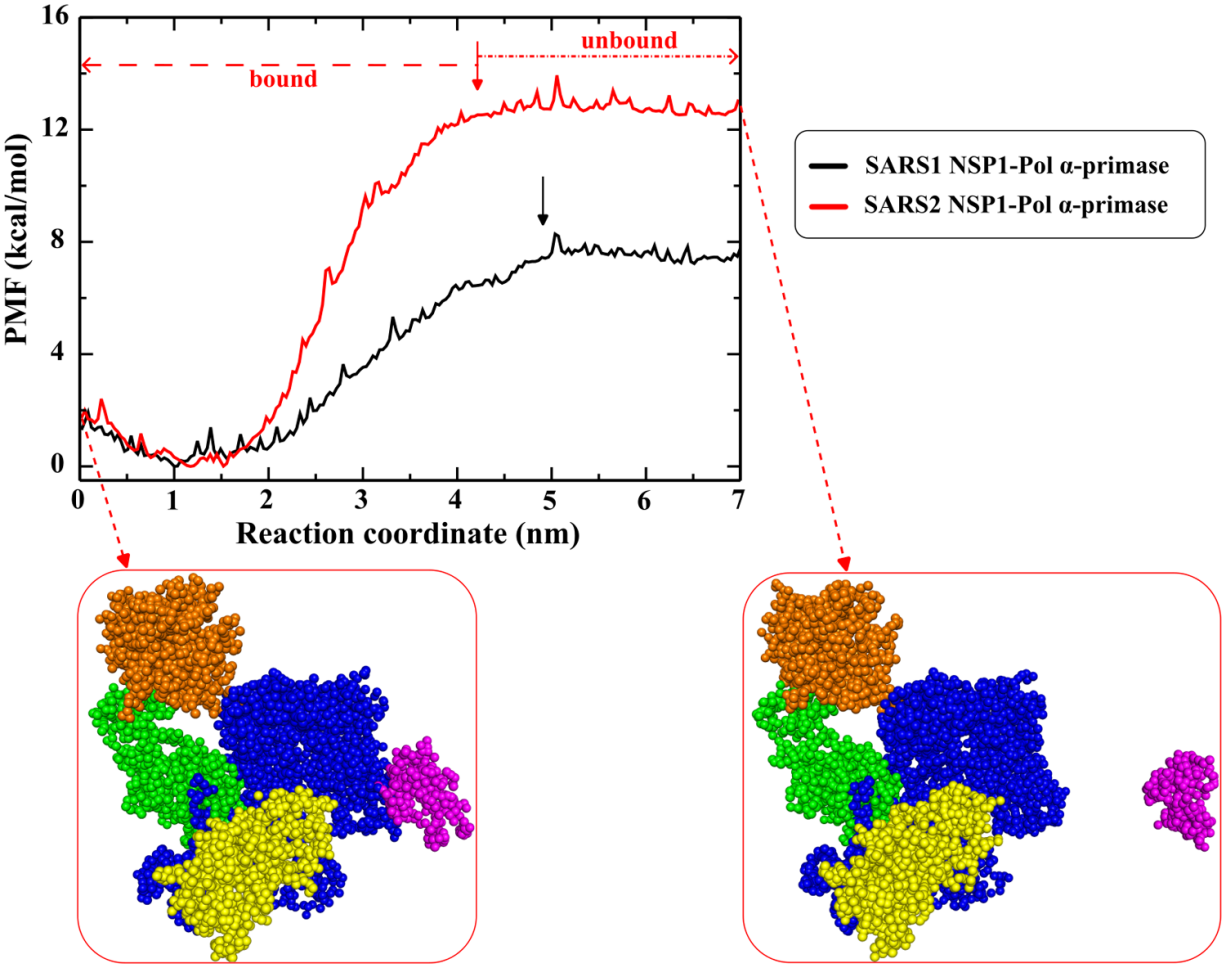
1D-PMF for a [50–1000 ns] time window as a function of the RC. The left and right snapshots refer to the bound and unbound state of SARS2 NSP1-Pol α-primase. The arrow indicates the position of the cutoff distance between bound and unbound states. These results were estimated from coarse-grained US simulations using the MARTINI model.

In detail, the Δ*G*_bind_ values for the binding of SARS-CoV-1 NSP1 and SARS-CoV-2 NSP1 to Pol α-primase correspond to −7.01 and −12.02 kcal/mol, respectively (Table 3). Our calculations indicate that the binding affinity of SARS-CoV-2 NSP1 to Pol α-primase appears to be much more stronger than that of SARS-CoV-1 NSP1. Here, the coarse-grained US results align with all-atom SMD findings on SARS-CoV-1 NSP1 and SARS-CoV-2 NSP1 binding to Pol α-primase. Furthermore, the experimental result indicated that the binding affinity of SARS-CoV-1 NSP1 to Pol α-primase, inhibiting the DNA replication process, is estimated at −6.82 kcal/mol,^30^ which is in good agreement with our computed binding energy for SARS1 NSP1-Pol α-primase. In summary, our analysis of both SARS1 NSP1-Pol α-primase and SARS2 NSP1-Pol α-primase highlights a crucial observation: SARS-CoV-2 poses a greater risk than SARS-CoV-1 in impeding DNA replication for DNA synthesis.

**Table 3:** Binding Free Energy (kcal/mol) of SARS-CoV-1 NSP1 and SARS-CoV-2 NSP1 to Pol α-Primase Estimated from the Experiment and Our Computations

	SARS1 NSP1-Pol α-primase	SARS2 NSP1-Pol α-primase
experiment^30^	**–6.82**	N/A
our computation	**–7.01**	**–12.02**

### 3.6 Binding of DNA to Pol α-Primase Is Reduced by Both SARS-CoV-1 NSP1 and SARS-CoV-2 NSP1, with SARS-CoV-2 NSP1 Exhibiting a More Pronounced Inhibitory Effect

As mentioned in previous parts, we hypothesized that SARS-CoV-1 NSP1 or SARS-CoV-2 NSP1 binds to Pol α-primase inhibiting the DNA synthesis process. This hypothesis can be supported by the observation that they bind to Pol α-primase, resulting in decreased binding between DNA and Pol α-primase. To test this, we constructed structures of SARS-CoV-1 NSP1 and SARS-CoV-2 NSP1 bound to Pol α-primase with DNA, as shown in Figure 6A. To estimate the free energy of DNA binding to Pol α-primase, as well as to SARS1 NSP1-Pol α-primase and SARS2 NSP1-Pol α-primase complexes, the MM-PBSA method (eq 5) was applied using snapshots recorded during the last 130 ns of the MD trajectories.

**Figure 6:**
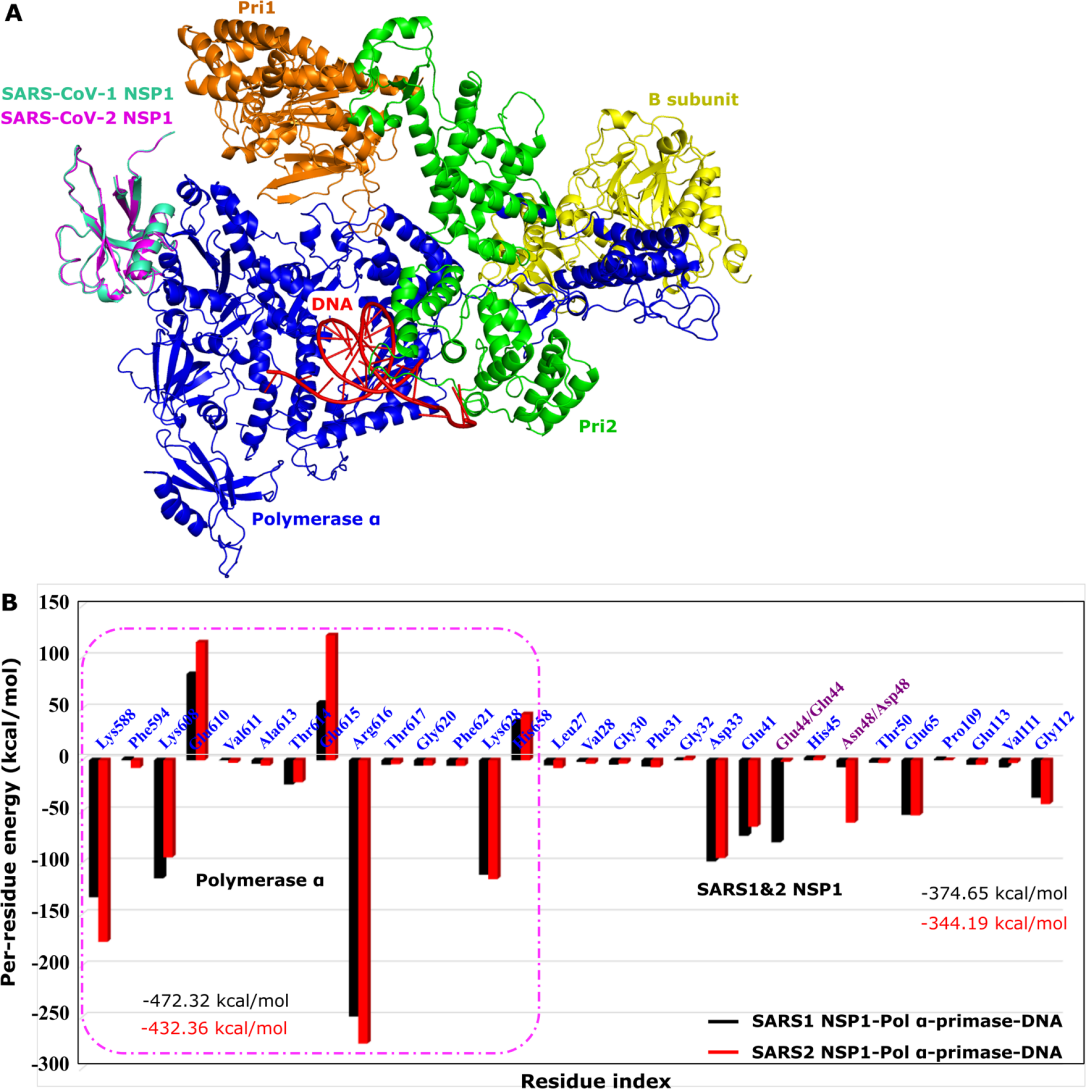
(A) 3D structure of SARS-CoV-1 NSP1 or SARS-CoV-2 NSP1 binds to Pol α-primase with DNA. (B) Total nonbonded interaction energy of per-residue at the binding regions of SARS1 NSP1-Pol α-primase-DNA (black) and SARS2 NSP1-Pol α-primase-DNA (red). The results were averaged from the last 130 ns of all-atom conventional MD runs.

The binding free energies (Δ*G*_MM‑PBSA_) of the three complexes are presented in Table 4. The electrostatic energy (Δ*E*_elec_) plays a more significant role than the vdW energy (Δ*E*_vdW_). Changes in the nonpolar solvation energy (Δ*E*_nonpolar_) and entropic term (−*T*Δ*S*) do not make a major contribution to the differences in binding between the three complexes. The loss of polar solvation energy (Δ*E*_polar_) is compensated by the remaining components of the binding free energy in these model systems. We found that Δ*G*_MM‑PBSA_ = −41.12 ± 3.16 kcal/mol for DNA binding to Pol α-primase was more negative compared to Δ*G*_MM‑PBSA_ = −32.89 ± 2.77 kcal/mol for the SARS1 NSP1-Pol α-primase-DNA complex and Δ*G*_MM‑PBSA_ = −28.47 ± 2.03 kcal/mol for the SARS2 NSP1-Pol α-primase-DNA complex. Thus, the order of binding strength is as follows: Pol α-primase-DNA < SARS1 NSP1-Pol α-primase-DNA < SARS2 NSP1-Pol α-primase-DNA, indicating that SARS-CoV-1 NSP1 and SARS-CoV-2 NSP1 bind to Pol α-primase and reduce the binding between DNA and Pol α-primase. This result suggests that in the presence of SARS-CoV-1 NSP1 or SARS-CoV-2 NSP1 reduces DNA binding to the Pol α-primase complex, thereby disrupting the DNA synthesis process and supporting our hypothesis. Furthermore, the weaker binding of DNA to the SARS2 NSP1-Pol α-primase complex compared to the SARS1 NSP1-Pol α-primase counterpart indicates that SARS-CoV-2 NSP1 inhibits DNA synthesis more effectively than SARS-CoV-1 NSP1.

**Table 4:** Binding Free Energy (kcal/mol) of DNA to Pol α-Primase, SARS1 NSP1-Pol α-Primase and SARS2 NSP1-Pol α-Primase Estimated Using the MM-PBSA Method

	Δ*E*_elec_	Δ*E*_vdW_	Δ*G*_polar_	Δ*G*_nonpolar_	–*T*Δ*S*	Δ*G*_MM‑PBSA_
Pol α-primase - DNA	–4143.38 ± 35.60	–246.13 ± 6.01	4309.50 ± 58.22	–25.63 ± 1.98	64.52 ± 5.77	**–41.12 ± 3.16**
SARS1 NSP1-Pol α-primase-DNA	–4005.61 ± 47.09	–180.67 ± 7.11	4113.45 ± 43.21	–25.98 ± 2.77	65.92 ± 6.56	**–32.89 ± 2.77**
SARS2 NSP1-Pol α-primase-DNA	–3782.91 ± 50.53	–219.35 ± 8.44	3930.98 ± 48.74	–24.12 ± 2.01	66.93 ± 6.13	**–28.47 ± 2.03**

We also calculated the average per-residue interaction energy over the last 130 ns at the binding site for each system to identify the key residues involved in the binding of SARS-CoV-1 NSP1/SARS-CoV-2 NSP1 to Pol α-primase with DNA (Figure 6B). In detail, the total interaction energy of all residues at the binding region for SARS1 NSP1-Pol α-primase-DNA (−374.65 kcal/mol for SARS-CoV-1 NSP1 and −472.32 kcal/mol for Pol α-primase) and SARS2 NSP1-Pol α-primase-DNA (−344.19 kcal/mol for SARS-CoV-2 NSP1 and −432.36 kcal/mol for Pol α-primase) is significantly different compared to SARS-CoV-1 NSP1/SARS-CoV-2 NSP1 binding to Pol α-primase without DNA (see Figure 3D). Specifically, the interaction energy of all residues in SARS-CoV-1 NSP1/SARS-CoV-2 NSP1 is approximately twice as large with DNA compared to the system without DNA, while the total interaction energy of all residues at the binding site of Pol α-primase remains similar between the DNA-bound and DNA-free states. Importantly, the total interaction energy of SARS-CoV-2 NSP1 at the binding site is higher than that of SARS-CoV-1 NSP1, which contrasts with the interaction energy observed in the absence of DNA. Here, most of the key residues are similar to those in the absence of DNA, with the exception of residues Glu615 and His658, whose contributions are significant in the presence of DNA but minimal in the absence of DNA.

## 4 Conclusions

We investigated the interaction of SARS-CoV-1 NSP1 and SARS-CoV-2 NSP1 with Pol α-primase. Our all-atom SMD findings reveal that SARS-CoV-2 NSP1 exhibits a stronger binding affinity to Pol α-primase compared to SARS-CoV-1 NSP1, which is also consistent with the results obtained from coarse-grained US simulations. These computational outcomes support the conclusion that SARS-CoV-2 poses a higher risk than SARS-CoV-1 in inhibiting DNA replication for DNA synthesis.

The stability of both SARS1 NSP1-Pol α-primase and SARS2 NSP1-Pol α-primase complexes is primarily attributed to electrostatic interactions. Additionally, the contribution of the charged residues from SARS-CoV-1 NSP1 and SARS-CoV-2 NSP1 is identified as a crucial factor influencing the binding affinity of these complexes to Pol α-primase. The hydrogen bond pair Asp33::Arg616 in SARS1 NSP1-Pol α-primase, and hydrogen bond pairs Asp33::Arg616 and Asp33::Lys655 in SARS2 NSP1-Pol α-primase, emerged as a key factor driving the interaction of both SARS-CoV-1 NSP1 and SARS-CoV-2 NSP1 with Pol α-primase. Importantly, Asp33 in SARS-CoV-2 NSP1 exhibits superior solubility and stability in its interaction with Pol α-primase compared to that of SARS-CoV-1 NSP1. While all-atom SMD simulations provide insights into the relative binding affinity, the Δ*G*_*bind*_ value determined from coarse-grained US calculations can be compared with experimental results. However, the all-atom SMD approach offers the advantage of allowing detailed characterization of the binding process at the atomic level, highlighting the importance of electrostatic interactions over van der Waals interactions in stabilizing the heterodimers studied.

The binding free energy of DNA to Pol α-primase, SARS1 NSP1-Pol α-primase, and SARS2 NSP1-Pol α-primase, estimated using the MM-PBSA method, follows the order: Pol α-primase-DNA < SARS1 NSP1-Pol α-primase-DNA < SARS2 NSP1-Pol α-primase-DNA. This indicates that the presence of either SARS-CoV-1 NSP1 or SARS-CoV-2 NSP1 reduces DNA binding to Pol α-primase, providing compelling evidence for the disruption of the DNA synthesis process.

## Supplementary Material



## Data Availability

The data can be downloaded from the DOI 10.5281/zenodo.15399000 (the link: https://zenodo.org/records/15399000?token=eyJhbGciOiJIUzUxMiJ9.eyJpZCI6IjdmZmY1NjRjLTU5Y2UtNGQ1NS04N2Y1LTY5Y2Y2YjllZTA0NSIsImRhdGEiOnt9LCJyYW5kb20iOiIyZjM2NzIwYzhhODRkZjBkYTA2ZmZhZTllN2U2ZDQwZSJ9.9DQ_Qk9OJEjYavmrwcD3auUiywC2wP4hjXFQxwOSnRQXNRyFwe-8C1UNVyQuELlVaxv0QtLlEgFiY7HfplOfzA).

## Notes

RMSD of the complexes (Figure S1); hydrogen bond networks and nonbonded contacts between SARS-CoV-1 NSP1 or SARS-CoV-2 NSP1 and DNA polymerase α (Figure S2); pulling directions used in the SMD simulations (Figure S3); determination of the cutoff distance separating bound and unbound states for the SARS1 NSP1-Pol α-primase and SARS2 NSP1-Pol α-primase (Figure S4); 1D-PMF profiles as a function of the reaction coordinate (RC) for the SARS-CoV-1 NSP1 and SARS-CoV-2 NSP1 to Pol α-primase complexes, computed over the time windows [50–500 ns], [50–800 ns], and [50–1000 ns] (Figure S5); total charge of SARS-CoV-1 NSP1, SARS-CoV-2 NSP1, DNA polymerase α, B subunit, Pri1, and Pri2 (Table S1); nonbonded interaction energies SARS1 NSP1-Pol α-primase and SARS2 NSP1-Pol α-primase complexes in the presence of solvent and averaged over the first 250 ps from 5 all-atom SMD trajectories (Table S2); and occupancy of hydrogen bond pairs averaged using a distance threshold increasing from 0.1 nm before to 0.15 nm after the force maximum (*F*_max_) in the SMD simulations (Table S3) (PDF)
