# Supplementary material for: Binding of SARS-CoV-1/2 NSP1 to DNA Polymerase α‑Primase Inhibits DNA Replication through Reduction of Interaction between DNA and DNA Polymerase α‑Primase
Source: J Chem Inf Model. 2025 Jul 30;65(15):8276–89. doi: 10.1021/acs.jcim.5c00999 (PMC12344767; doi:10.1021/acs.jcim.5c00999)
Supplement: Supplementary file 1 [file ci5c00999_si_001.pdf]

Supporting Information

Binding of SARS-CoV-1/2 NSP1 to DNA Polymerase α-Primase Inhibits DNA Replication through Reduction of Interaction between DNA and DNA Polymerase α-Primase

Hung Van Nguyen [1,*], **Nguyen Le Ngoc Lan** [2], and **Mai Suan Li** [1,*]

[1]*Institute of Physics, Polish Academy of Sciences, al. Lotnikow 32/46, 02-668, Warsaw, Poland.*
[2]*Biomedical Engineering Department, University of Technology - VNU HCM, 268 Ly Thuong Kiet Street, Ward 14, District 10, 740500, Ho Chi Minh City, Viet Nam.*

[*]Corresponding Authors: hungkhtn2008@gmail.com (HVN), and masli@ifpan.edu.pl (MSL)

Supporting Figures

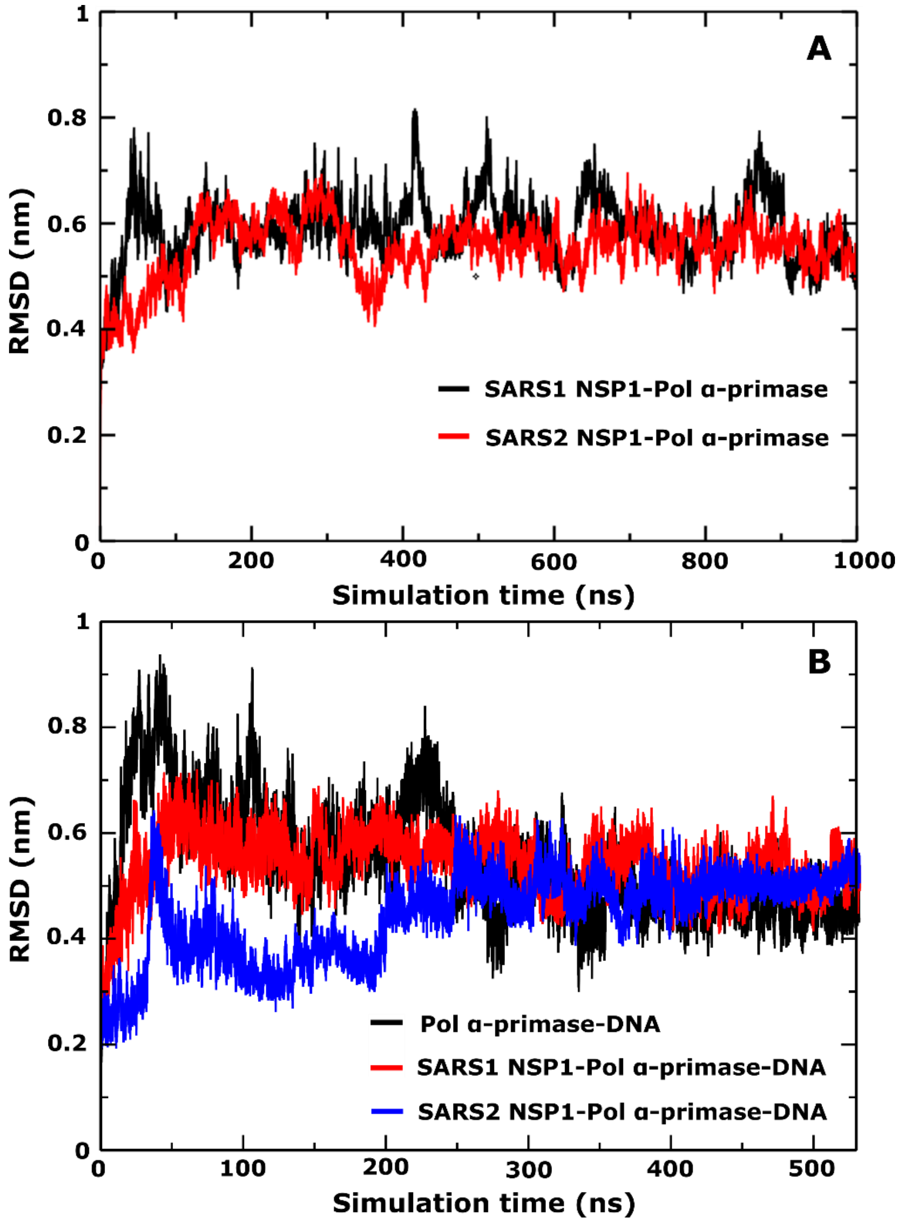

Figure S1: A) Root mean square deviation (RMSD) as a function of simulation time of SARS1 NSP1-Pol α-primase (black), and SARS2 NSP1-Pol α-primase (red) complexes. The results are obtained from 1000 ns conventional MD simulations. B) RMSD as a function of simulation time of Pol α-primase-DNA (black), SARS1 NSP1-Pol α-primase-DNA (red), and SARS2 NSP1-Pol α-primase-DNA (blue) complexes. The results are obtained from 530 ns conventional MD simulations.

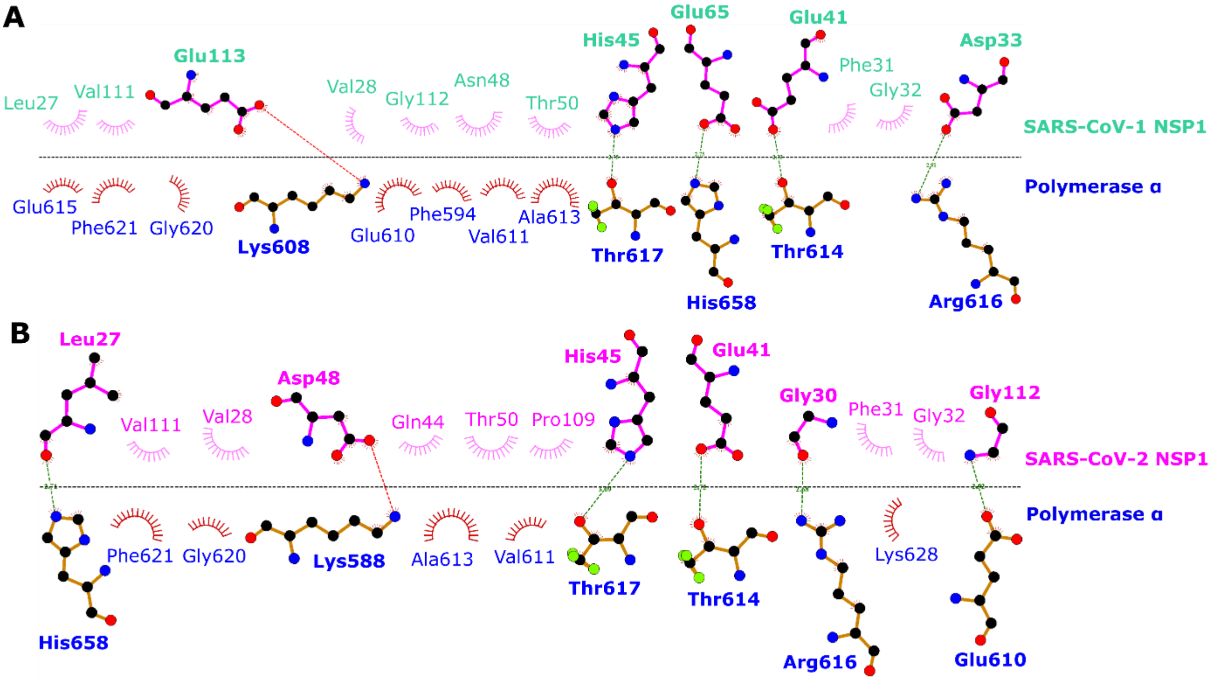

Figure S2: A) Networks of hydrogen bond and non-bonded contacts between SARS-CoV-1 NSP1 and DNA polymerase α. B) The same as in A but for the complex of SARS-CoV-2 and DNA polymerase α. The dotted line refers to HB, while the "eye" refers to NBC. The analyzed structures are taken from the most populated structure obtained by clustering snapshots of 1000 ns conventional MD simulations for the SARS1 NSP1-Pol α-primase and SARS2 NSP1-Pol α-primase complexes.

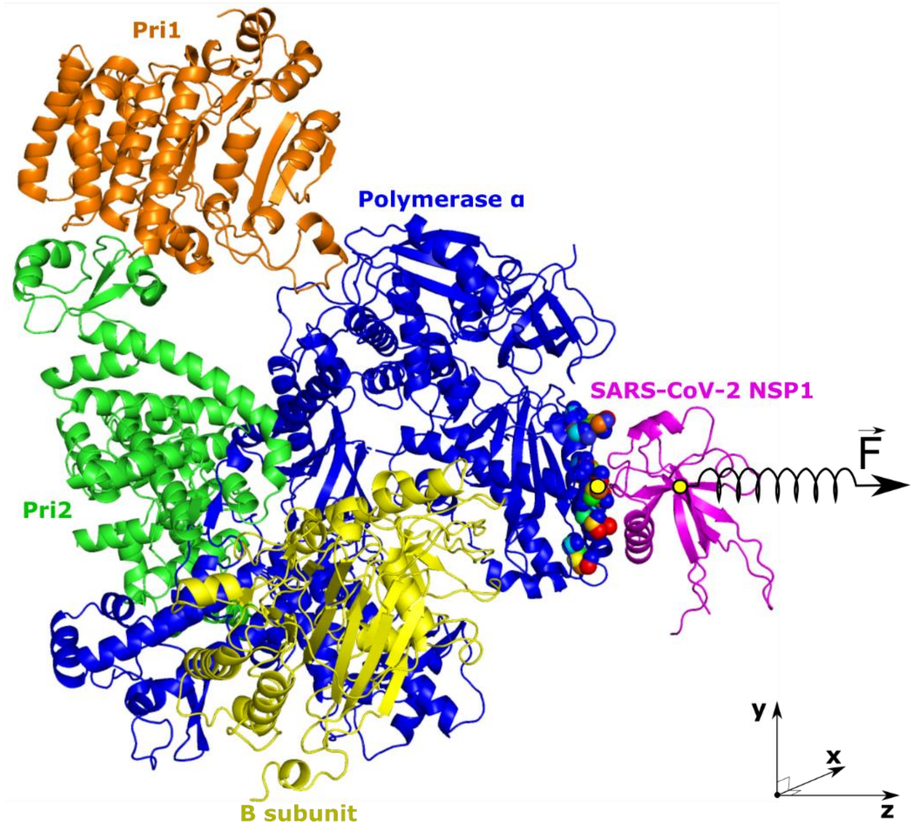

Figure S3: Structure of SARS-CoV-2 NSP1 bound to Pol α-primase (including DNA polymerase α, B subunit, Pri1, Pri2). External force is applied to SARS-CoV-2 NSP1 through a dummy atom connected to a spring. The pulling direction in SMD simulations is shown with a spring along the z-axis.

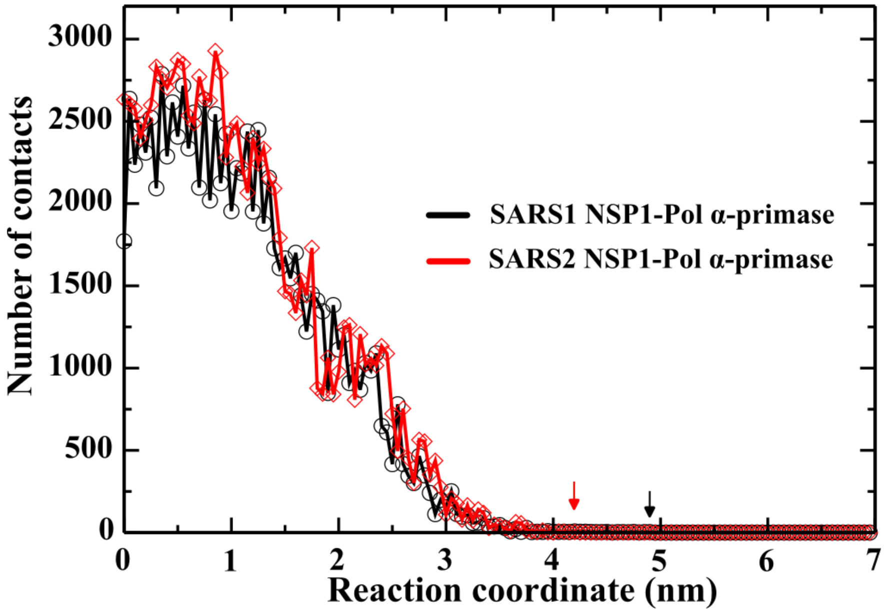

Figure S4: Determination of the cutoff distance separating bound and unbound states for SARS1 NSP1-Pol α-primase (black arrow) and SARS2 NSP1-Pol α-primase (red arrow) complexes. Dependence of the number of interchain contacts on the RC in the coarse-grained US simulation. The number of contacts was calculated using the last snapshot of the 1000 ns trajectory for each coarse-grained US window.

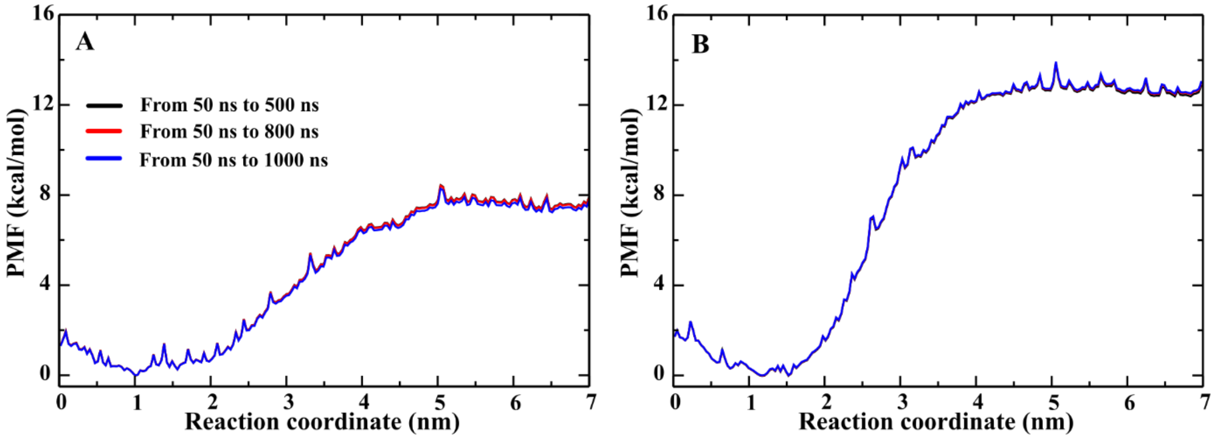

Figure S5: 1D-PMF as a function of the RC for A) SARS1 NSP1-Pol α-primase, and B) SARS2 NSP1-Pol α-primase obtained for time windows of [50-500 ns], [50-800 ns], and [50-1000 ns]. These results were obtained from coarse-grained US simulations using the MARTINI model.

Supporting Tables

Table S1: Total charge of the SARS-CoV-1 NSP1, SARS-CoV-2 NSP1, DNA polymerase α, B subunit, Pri1 and Pri2.

**Structure**	**Total charge (e)**
SARS-CoV-1 NSP1	-2e
SARS-CoV-2 NSP1	-2e
DNA polymerase α	+5e
B subunit	-14e
Pri1	+9e
Pri2	+3e

Table S2: Non-bonded interaction energies (kcal/mol) of SARS1 NSP1-Pol α-primase and SARS2 NSP1-Pol α-primase complexes. The results were obtained in the presence of solvent and averaged over the first 250 ps from 5 all-atom SMD trajectories. The errors represent standard deviations.

	SARS1 NSP1-Pol α-primase	SARS2 NSP1-Pol α-primase
ΔE_{vdW}	-237.98±1.94	-253.24±1.17
ΔE_{elec}	-3325.17±14.12	-3353.68±13.92
ΔE_{total}	-3563.15±16.06	-3606.92±15.09

Table S3: Occupancy of hydrogen bond pairs averaged from a distance threshold of 0.1 nm before to 0.15 nm after F_{max}, according to the displacement-time profile of SARS1 NSP1-Pol α-primase and SARS2 NSP1-Pol α-primase, for 5 trajectories of all-atom SMD simulations.

Complexes	Trajectories	Hydrogen bond pairs (NSP1::Pol α-primase)	Occupancy (%)
SARS1 NSP1-Pol α-primase	Trajectory 1	**Asp33::Arg616**	**87.22**
		Glu65::Arg616	6.44
		Glu41::Thr614	1.14
		Asp25::Lys628	0.28
		Arg24::Lys628	4.83
		Leu27::Lys625	0.28
		Leu27::Phe621	1.42
		Glu65::Pro657	0.28
		Glu65::His658	5.97
		Asp33::Lys655	3.98
	Trajectory 2	**Asp33::Lys655**	**74.27**
		**Glu65::His658**	**40.04**
		Asp33::Arg616	7.42
		Gly32::Arg616	1.12
		Leu27::Phe621	2.68
	Trajectory 3	**Asp33::Arg616**	**21.32**
		**Asp25::Lys628**	**20.81**
		Leu27::Phe621	0.51
		Arg24::Lys628	5.58
		Asn48::Lys599	1.52
		Val28::Thr617	0.51
	Trajectory 4	**Asp33::Arg616**	**20.55**
		Glu65::His658	8.60
		Asp25::Lys628	0.84
		Val111::Phe621	6.92
		Leu27::Phe621	0.42
		Glu65::Arg616	0.84
		Asn48::Lys599	6.50
		Leu27::Thr617	0.42

	Trajectory 5	**Asp33::Arg616**	**62.10**
		**Asp25::Lys628**	**24.65**
		Glu65::His658	6.42
		Asp33::Lys655	2.14
		Gly32::Arg616	0.21
		Glu65::Pro657	0.64
		Leu27::Phe621	0.64
		Glu113::Lys628	0.21
		Arg24::Lys628	0.64
SARS2 NSP1-Pol α-primase	Trajectory 1	**Asp33::Arg616**	**67.26**
		**His45::Thr617**	**38.49**
		**Asp33::Lys655**	**43.81**
		His45::Thr614	7.74
		His45::Ala613	1.99
		Glu65::Pro657	0.44
		Val111::Glu610	0.44
		Asp48::Phe594	1.33
		Gly112::Glu610	1.99
		Glu41::Thr614	2.88
		Thr50::Ala613	0.66
		Asp48::Lys588	0.88
		Val28::Thr617	0.66
		Leu27::Phe621	0.88
		Phe31::Arg616	0.22
		Glu65::His658	0.44
	Trajectory 2	**Asp33::Lys655**	**48.67**
		**Asp33::Arg616**	**82.96**
		Glu65::His658	0.44
		Phe31::Arg616	0.22
		Gly32::Arg616	0.66
		Leu27::Phe621	1.54
		His45::Ala613	0.66
		His45::Thr614	0.44
		Val111::Phe621	0.44
		Leu27::Lys628	0.22
		Gly112::Glu610	0.22
	Trajectory 3	**Asp33::Lys655**	**71.73**
		**Asp33::Arg616**	**96.77**
		Val28::Thr617	1.90
		Leu27::Phe621	0.57
		Gly32::Arg616	0.38
		Glu65::His658	3.42
		Gly112::Glu610	2.66

		Val111::Phe621	0.76
		Phe31::Arg616	3.61
		Arg24::Lys628	3.98
		Glu65::Pro657	0.19
		His45::Ala613	1.14
		Val111::Glu610	0.19
	Trajectory 4	**Asp33::Lys655**	**66.53**
		**Asp33::Arg616**	**97.91**
		Glu65::His658	0.14
		His45::Thr614	0.28
		Val28::Thr617	2.93
		Val111::Phe621	0.14
		Gly112::Glu610	0.14
		Asp25::Lys628	1.39
		Arg24::Lys628	0.14
		Leu27::Phe621	0.70
		Glu65::Pro657	0.42
		Val28::Gly620	0.14
		Val111::Val611	0.56
		Phe31::Arg616	0.14
	Trajectory 5	**Asp33::Lys655**	**26.42**
		**Asp33::Arg616**	**68.47**
		**Arg24::Lys628**	**28.98**
		Glu41::Thr614	0.28
		Phe31::Arg616	0.57
		Val111::Phe621	0.57
		Val28::Thr617	0.57
		Leu27::Phe621	0.85
		Glu65::Pro657	0.57
